# Patterns and drivers of the distribution of *Leishmania* infection in humans using environmental and socioeconomic factors: a modeling study in mainland Portugal

**DOI:** 10.1186/s13071-026-07451-9

**Published:** 2026-05-22

**Authors:** Rafael Rocha, César Capinha, Carla Maia

**Affiliations:** 1 Global Health and Tropical Medicine (GHTM), Associate Laboratory in Translation and Innovation Towards Global Health, LA-REAL, Instituto de Higiene e Medicina Tropical (IHMT), Universidade Nova de Lisboa (UNL), Lisbon, Portugal; 2 Centre of Geographical Studies, Institute of Geography and Spatial Planning, University of Lisbon, Lisbon, Portugal; 3 Associate Laboratory TERRA, Lisbon, Portugal

**Keywords:** Climate, Distribution, *Leishmania*, One Health, Portugal, Seroprevalence, Social determinants of health, Spatial modeling, Visceral leishmaniasis

## Abstract

**Background:**

Leishmaniasis remains endemic in the Mediterranean region, where *Leishmania infantum* causes both visceral disease and widespread asymptomatic infection. In Portugal, the true burden and geographical distribution of human infection are incompletely understood and likely influenced by environmental, climatic, and socioeconomic factors. This study aimed to identify determinants of symptomatic and asymptomatic *Leishmania* infection, map current distribution, and explore spatial heterogeneity across mainland Portugal.

**Methods:**

We performed a municipality-level ecological modeling study using two complementary datasets: notified visceral leishmaniasis (VL) cases reported between 2010 and 2020 and *Leishmania* seroprevalence among blood donors in 2022. Environmental, climatic, social, and economic covariates were extracted from national and international databases. Zero-inflated beta-regression models with random intercepts were fitted to assess associations while accounting for excess zeros and regional clustering.

**Results:**

Marked spatial heterogeneity was observed for both VL incidence and *Leishmania* seroprevalence. Higher VL incidence was independently associated with greater migrant population share and a higher proportion of residents whose highest attained education was basic education or below, while greater forest cover and a proxy for stray animals were inversely associated. In contrast, seroprevalence was higher in municipalities with a greater proportion of residents living in small localities and lower altitude and precipitation and was inversely associated with lower educational attainment and stray-animal proxies. Climatic and socioeconomic factors showed differential associations with clinical disease versus asymptomatic infection.

**Conclusions:**

Distribution of human VL and *Leishmania* infection in Portugal reflects a complex interaction between environmental suitability, social vulnerability, and surveillance context. Integrating social determinants into One Health-based control strategies and strengthening surveillance are essential to address current transmission patterns and anticipate future changes under climate change.

**Graphical Abstract:**

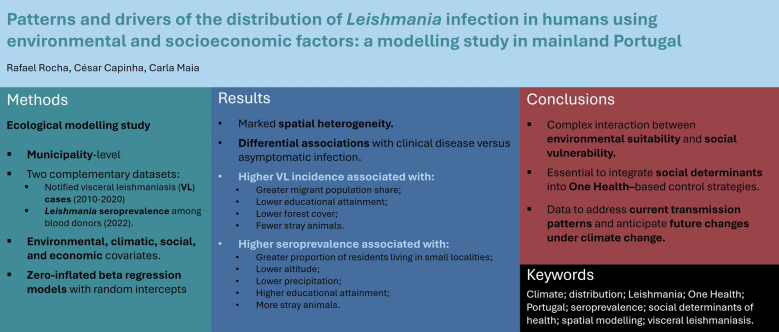

**Supplementary Information:**

The online version contains supplementary material available at 10.1186/s13071-026-07451-9.

## Background

Leishmaniases are a group of diseases caused by protozoan parasites of the genus *Leishmania*. These parasites are transmitted by phlebotomine sand flies, and the disease is zoonotic in most settings [1]. Clinical spectrum of symptomatic disease is usually grouped into two main syndromes, visceral leishmaniasis (VL) and cutaneous leishmaniasis (CL) [1], both of which are endemic and geographically widespread in the Mediterranean region. In this region, *L. infantum*, which belongs to the *L. donovani* complex, is the etiologic species of most autochthonous human leishmaniasis cases [2]. Although infection by species of the *Leishmania donovani* complex is mostly recognized by its symptomatic presentations, many studies in *L. donovani* complex endemic areas suggest asymptomatic infection is the most common outcome of exposure to the parasite [3–5]. Understanding the distribution of asymptomatic infections can contribute decisively to improving updated epidemiologic knowledge and guide control strategies.

In the western Mediterranean regions where *L. infantum* is endemic, including in Portugal, *Phlebotomus perniciosus* is the main vector [6] and dogs are the main reservoir for human infection [7]. Increasing evidence suggests that cats [8] and some wild animals (such as leporids [9]) may also play a relevant epidemiological role.

In the period from 2005 to 2020, 5813 VL cases were reported to the World Health Organization (WHO) in the European region, corresponding to an annual incidence of 0.03–0.21 per 100,000 population in this region, although in some countries the annual incidence exceeded 0.40 per 100,000 population, such as Albania, Montenegro, Malta, Greece, Spain, and North Macedonia [10]. In Portugal, reporting of VL cases to central public health authorities is mandatory, as part of a passive surveillance system. The most recent findings from this system showed that 6–14 cases were reported per year between 2014 and 2018, corresponding to an annual incidence of 0.06–0.14 cases per 100,000 population [11]. However, this likely represents a significant under-reporting of cases, as revealed in previous studies where only 38.6% (between 1999 and 2009) and 49.7% (between 2010 and 2020) of cases diagnosed in public hospitals were notified to central public health authorities [12, 13]. In addition, a nationwide serological study conducted among blood donors estimated a true seroprevalence of 4.8%, with significant regional variation [14].

In the Mediterranean region, some modeling studies have produced predictions of current and future distribution of several *Phlebotomus* sand fly species, projecting a northward expansion of areas with suitable climatic conditions for most species [15–17]. In accordance with that, a recent Europe-wide modeling study demonstrated that climatic suitability for *L. infantum* transmission has increased over the past two decades [18].

In the Iberian Peninsula, a recent study combined the potential distribution of *P. perniciosus* in the Iberian Peninsula and the calculation of the infection rate of the parasite in the vector to model the risk of contracting the disease in dogs [19]. Modeling has also been used to predict the distribution of canine leishmaniasis in western Europe on the basis of environmental variables [20]. However, presence of leishmaniasis cases in a human population is not fully explained only by the presence of infected vectors and the main hosts and likely depends on additional environmental and human factors, including social and economic determinants.

Therefore, this study aimed to: (i) search for associations between the current known geographical data of *Leishmania* infection (symptomatic and asymptomatic) in humans in mainland Portugal and environmental, social, and economic factors; and (ii) predict the current distribution of leishmaniasis in mainland Portugal.

## Methods

### Study population

Mainland Portugal is located in Southwest Europe, bordering Spain and the Atlantic Ocean, and is divided for statistical purposes into seven Nomenclature of Territorial Units for Statistics (NUTS)2 regions, 24 NUTS3 regions [21], and 278 municipalities. According to the 2021 national census, the population of mainland Portugal was 9,857,593 inhabitants [22].

This study focused on two distinct populations in mainland Portugal: people diagnosed with VL between 2010 and 2020 and blood donors with serological evidence of exposure to *Leishmania* parasites in 2022.

### Data collection

#### Human visceral leishmaniasis cases

Information on people diagnosed with VL in Portugal is reported to the Directorate-General of Health (Direção Geral de Saúde [DGS]) through a compulsory notification system. Mandatory notifications of VL cases, initially done in paper format, have, since 2014, been submitted through an electronic platform, the National Epidemiologic Surveillance System (SINAVE) [23].

Data on reported VL cases were obtained from a previously conducted study [13], in which the DGS was contacted and access to notified cases of VL between 2010 and 2020 was requested. A total of 114 VL cases were reported in this period. Sociodemographic and clinical data of these cases were provided by the DGS in a codified database. Prior to analysis, data cleaning to identify potential inconsistencies was performed, including duplicate records, missing values, and implausible entries. Records with incomplete key variables (e.g., municipality of residence) were excluded, and consistency checks were applied across time and location variables to ensure coherence of the dataset.

#### Human seroprevalence in blood donors

Data from blood donors were originally collected as part of a separate cross-sectional *Leishmania* seroprevalence study [14]. A detailed description of the methodology is available in ref. [14], with a summary presented in Supplementary Table 1. That study focused on the population of people who donate blood in mainland Portugal, recruited through the Portuguese Institute of Blood and Transplantation (IPST) and the Immunohemotherapy Departments (IHDs) of public hospitals in the Alentejo and Algarve regions. Both the IPST and IHDs conduct regular blood collections at fixed centers and mobile units operating in rural and urban areas. In 2022, these institutions oversaw over 190,000 blood donations [24], following a rigorous and standardized triage process.

The sampling process was stratified by municipality. Individuals participating in the original study attended one of the collaborating institutions between February and June 2022 and were deemed eligible for blood donation. Only individuals aged 18–65 years were included. Participant enrollment took place during non-randomly selected blood collection sessions, but within each session, invitations to participate were issued randomly, on the basis of the time of arrival at the blood collection center or unit. Participants completed a self-administered structured paper questionnaire addressing sociodemographic factors.

From the routinely collected blood samples, 1.5 mL of serum was used to detect antileishmanial antibodies, using enzyme-linked immunosorbent assay (ELISA) (*Leishmania* ELISA IgG + IgM, Vircell^®^, Spain), following the manufacturer’s instructions and cutoffs. The sensitivity and specificity of the ELISA, according to the manufacturer, are 97% and 99%, respectively. A single determination was performed for each serum sample. Samples were classified as positive, negative, or borderline (when optical density was < 10% lower or higher than the average value of the borderline controls).

The sample size effectively obtained was 3783 individuals. Of these, 3617 gave their consent for their data to be used in subsequent studies. All these individuals were included in the present modeling study.

#### Environmental, social, and economic data

Environmental, social, and economic data were extracted at the municipality level from publicly available national and international databases. The following variables were collected from the 2021 national census [22]: population density (inhabitants/km^2^); percentage of male sex resident population; percentage of resident population aged 0–14 years old; percentage of resident population living in localities with under 5000 inhabitants; percentage of migrant population; percentage of resident population with basic education or lower; and percentage of unemployed resident population. Data on purchasing power per capita in 2021 was extracted from the Pordata database [25]. Average altitude was obtained from the Portuguese Directorate-General for Territory (Direção-Geral do Território) [26]. Climatic variables, including average minimum winter temperature, average maximum summer temperature, and average annual precipitation were extracted from the Climatologies at High Resolution for the Earth’s Land Surface Areas (CHELSA) database [27]. Land-use characteristics, expressed as the proportion of forested or agricultural area, were derived from the Land Use and Land Cover Map (Carta de Uso e Ocupação do Solo) provided by the Direção-Geral do Território [28]. Finally, data on stray animals were obtained from the National Census of Stray Animals [29] and categorized according to the average annual number of stray animals collected between 2017 and 2021.

### Statistical analysis

Mean annual incidence of VL in 2010–2020, in adults and in children (at municipality level), was estimated on the basis of the following formula: Incidence in region = ((New cases in 2010–2020) / (At-risk population × Timeframe)) × 100,000, considering a timeframe of 11 years and an at-risk population, for each region, consisting of the arithmetic mean between the population size estimated in the National Census of 2011 and 2021, according to the National Institute of Statistics [22].

Estimated prevalence of anti-*Leishmania* antibodies in adult blood donors in 2022 (at municipality level) was obtained from a previous study [14], in which prevalence was calculated using the following formula: Prevalence = ((number of positive or borderline individuals) / (number of sampled individuals)) × 100. Geographical representation of estimated incidence and prevalence at municipality level was carried out using QGIS^®^ Version 3.40.

To quantify associations between municipality-level outcomes and environmental/socioeconomic covariates while accounting for geographical clustering, we fitted generalized linear mixed models (GLMMs) separately for (i) mean annual visceral leishmaniasis (VL) incidence at municipality level (2010–2020) and (ii) municipality-level seroprevalence of anti-*Leishmania* antibodies among blood donors (2022) [30]. Both outcomes were treated as bounded continuous measures and rescaled to the (0–1) interval (outcome/100) to allow modeling using beta-regression, which is appropriate for proportion-type data and accommodates skewness and heteroskedasticity. Because many municipalities presented zero values (75% for incidence and 51% for seroprevalence), we used zero-inflated beta-regression models, which combine a beta-distributed component for nonzero values with a point-mass component to accommodate excess zeros [31].

Models were fitted in R using the glmmTMB package, specifying a beta family with a constant zero-inflation term [32]. Formally, the outcome ${Y}_{i}$ was assumed to follow a mixture of a point mass at zero and a beta distribution for $0<{Y}_{i}<1$, with $logit({\mu}_{i})={X}_{i}\beta +{u}_{j[i]}$ for the conditional mean and $logit({\pi}_{i})={\gamma}_{0}$ for the zero-inflation component. Exponentiated coefficients (exp(*β*)) from the conditional component represent multiplicative effects on the odds of the mean proportion (logit scale), while those from the zero-inflation component are interpreted as odds ratios for the probability of structural zeros. Fixed effects included: population density, percentage male, percentage aged 0–14 years, percentage living in small localities, percentage migrants, percentage with basic education or lower, unemployment percentage, purchasing power per capita, altitude, minimum winter temperature, maximum summer temperature, annual precipitation, percentage agricultural area, percentage forest area, and a proxy for stray animals. To account for regional clustering, we included a random intercept for the NUTS3 region (1|NUTS3). Residual spatial autocorrelation was assessed using simulation-based diagnostics implemented in the DHARMa package, on the basis of municipality centroid coordinates (longitude and latitude). This approach provides a Moran’s *I*-type test of spatial dependence in model residuals. To reduce multicollinearity, candidate predictors with variance inflation factor (VIF) > 10 were excluded prior to model fitting [33]. For the seroprevalence model, municipalities with 100% seroprevalence were recoded to 99.9% to avoid boundary values at 1, which are not supported by the beta-component [31].

The model’s uncertainty was quantified using model-based simulations using the simulate() function of glmmTMB. For each municipality, 1000 simulated response values were generated using the model’s estimated parameters, reflecting both fixed and random effects as well as the zero-inflation component. These simulations produced a distribution of predicted values, from which 95% prediction intervals were derived. The standard deviation of simulated predictions was used to compute the coefficient of variation (CV), defined as the ratio of the standard deviation to the mean predicted value.

## Results

**Fig. 1:**
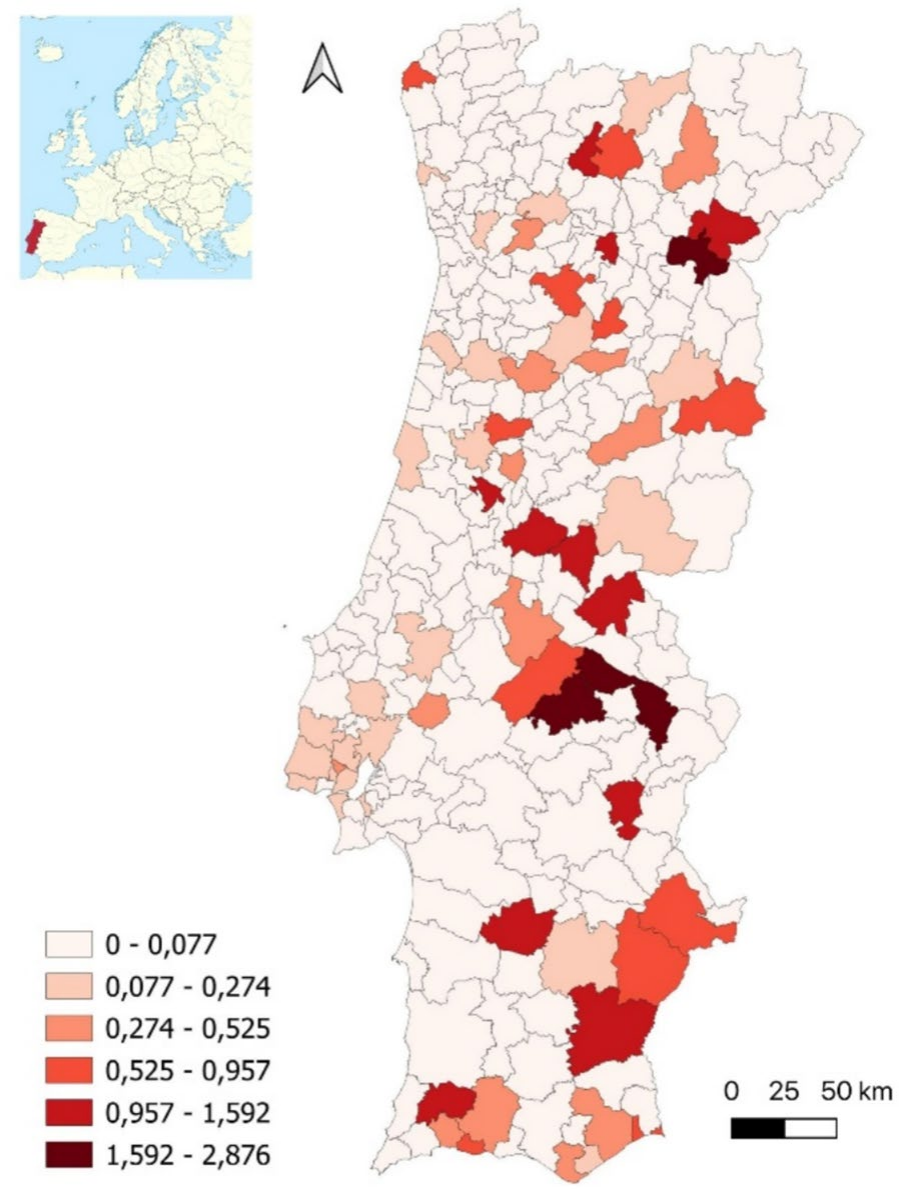
Estimated mean annual incidence of visceral leishmaniasis at the municipality level for the period 2010–2020

**Fig. 2:**
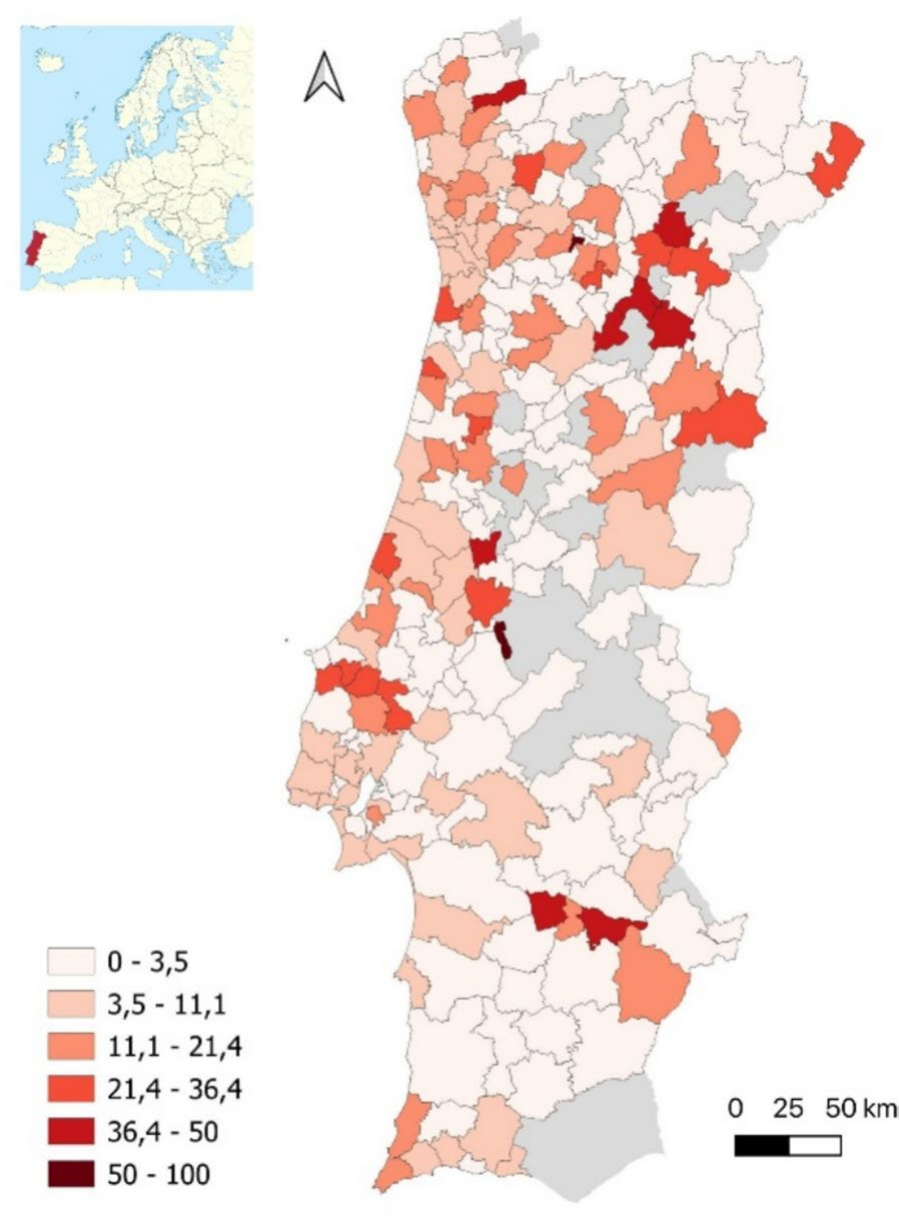
Estimated prevalence of anti-*Leishmania* antibodies among adult blood donors in 2022, mapped at the municipality level (adapted from [14])

The estimated mean annual incidence of visceral leishmaniasis at the municipality level for the period 2010–2020 is shown in Fig. 1, illustrating the spatial heterogeneity of reported cases across mainland Portugal. The estimated prevalence of anti-*Leishmania* antibodies among adult blood donors in 2022, also mapped at the municipality level, is shown in Fig. 2. The geographical distribution of the social, economic, environmental, and climatic variables included in the modeling analysis is shown in Supplementary Figs. 1 and 2.

### Regression results

In the model of municipality-level mean VL incidence (2010–2020), excess zeros were observed, and the zero-inflation component indicated a high baseline probability of structural zero incidence. In the conditional component, a higher proportion of migrants (exp(*β*) = 1.077, 95% confidence interval (CI): 1.020–1.137) and a higher proportion of residents with basic education or lower (exp(*β*) = 1.085, 95% CI: 1.031–1.141) were associated with increased mean incidence. In contrast, greater forest cover (exp(*β*) = 0.990, 95% CI: 0.982–0.997) and higher numbers of stray animals collected annually (exp(*β*) = 0.882, 95% CI: 0.805–0.966) were associated with lower mean incidence. The remaining covariates showed no clear evidence of association (Table 1; Supplementary Table 1). Residual spatial autocorrelation was weak (Moran’s *I* ≈ 0.007; *P* = 0.048), with a very small magnitude of spatial dependence, suggesting limited residual spatial structure. Prediction uncertainty, expressed as the coefficient of variation, ranged from 1.3 to 3.1, and showed a heterogeneous, spatially clustered distribution (Supplementary Fig. 3a).

**Table 1:** Association between municipal-level characteristics and mean visceral leishmaniasis incidence (2010–2020) estimated using zero-inflated beta-generalized linear mixed models

Variable	Effect	CI	*P* value
Intercept	0.0004	< 0.001–4.508	0.1
Intercept—zero inflation component	3	2.285–3.94	< 0.001
Population density	1	1–1	0.566
Percentage male sex	0.988	0.829–1.177	0.892
Percentage aged 0–14 years old	1.041	0.949–1.142	0.394
Percentage living in localities with < 2000 inhabitants	1.006	0.999–1.012	0.095
Percentage migrant	1.077	1.02–1.137	0.007
Percentage basic education or lower	1.085	1.031–1.141	0.002
Percentage unemployed	0.975	0.89–1.068	0.583
Purchasing power per capita	1.005	0.989–1.021	0.557
Average altitude	0.999	0.998–1.001	0.305
Average maximum summer temperature	1.018	0.905–1.146	0.765
Average precipitation	0.999	0.994–1.003	0.56
Percentage agricultural area	0.99	0.977–1.003	0.118
Percentage forested area	0.99	0.982–0.997	0.009
Stray animals collected annually (2017–2021)	0.882	0.805–0.966	0.007

Results are presented as exponentiated coefficients (exp(*β*)) with 95% confidence intervals (CI). For the zero-inflation component, exponentiated coefficients represent odds ratios for excess zeros. Variables with 95% CI not including 1 were considered to show evidence of association

In the model of blood-donor seroprevalence (2022), there was no clear evidence of excess structural zeros beyond the beta-component, as the zero-inflation intercept showed no meaningful deviation from 1 (exp(*β*) = 1.034, 95% CI: 0.801–1.335). Appreciable regional clustering remained, as indicated by the NUTS3 random intercept. In the conditional component, seroprevalence was positively associated with the proportion of residents living in small localities (< 2000 inhabitants) (exp(*β*) = 1.031, 95% CI: 1.020–1.042). In contrast, seroprevalence was negatively associated with the proportion of residents aged ≥ 14 years with basic education or lower (exp(*β*) = 0.929, 95% CI: 0.871–0.990), altitude (exp(*β*) = 0.997, 95% CI: 0.995–0.999), annual precipitation (exp(*β*) = 0.992, 95% CI: 0.984–0.999), and the number of stray animals collected annually (exp(*β*) = 0.873, 95% CI: 0.775–0.984). The remaining predictors showed no clear evidence of association (Table 2; Supplementary Table 2). Residual spatial autocorrelation was statistically significant (Moran’s *I* = 0.025; *P* < 0.001), although the magnitude of spatial dependence was relatively small. Prediction uncertainty (coefficient of variation: 0.8–3.7) was generally low to moderate and showed a heterogeneous, spatially clustered distribution (Supplementary Fig. 3b).

**Table 2:** Municipality-level determinants of *Leishmania infantum* seroprevalence among blood donors (2022) estimated using zero-inflated beta generalized linear mixed models.

Variable	Effect	CI	*P* value
Intercept	3.58 × 10^8^	57.4–2.24 × 10^15^	0.014
Zero inflation intercept	1.034	0.801–1.335	0.795
Population density	1.000	1.000–1.000	0.989
Percentage male	0.835	0.622–1.121	0.23
Percentage aged 0–14 years old	0.828	0.674–1.018	0.073
Percentage living in localities < 2000 inhabitants	1.031	1.020–1.042	< 0.001
Percentage migrant	0.953	0.882–1.030	0.222
Percentage with basic education or lower	0.929	0.871–0.990	0.024
Percentage unemployed	1.088	0.948–1.249	0.229
Purchasing power per capita	0.986	0.965–1.008	0.211
Average altitude	0.997	0.995–0.999	0.002
Average maximum summer temperature	1.024	0.867–1.209	0.781
Average precipitation	0.992	0.984–0.999	0.036
Percentage agricultural area	0.992	0.973–1.012	0.449
Percentage forested area	0.995	0.979–1.012	0.59
Stray animals collected annually (2017–2021)	0.873	0.775–0.984	0.026

Results are presented as exponentiated coefficients (exp(*β*)) with 95% confidence intervals (CI). For the zero-inflation component, exponentiated coefficients represent odds ratios for excess zeros. Variables whose 95% CI does not include 1 are considered to show evidence of association

## Discussion

This work identified key environmental, social, and economic factors associated with the distribution of leishmaniasis in mainland Portugal and characterized current spatial patterns of disease. The findings reveal complex and sometimes counterintuitive relationships between risk factors and disease transmission that warrant careful interpretation.

One of the most striking findings was the inverse relationship between stray dog populations and human VL incidence and blood donor prevalence; areas with fewer stray dogs paradoxically showed higher human disease/infection rates. This unexpected association can be explained through two complementary mechanisms.

First, effective stray dog control programs may reduce the size of the canine reservoir by removing dogs that are often highly exposed and potentially more vulnerable to clinical disease owing to poor nutrition and co-infections [33, 34]. In addition, *Leishmania*-infected dogs, in particular, are significantly more attractive to female sand flies than uninfected dogs [36, 37]. Since sand flies are generally opportunistic feeders, a reduction in the overall and the infected dog population may increase opportunistic biting on humans by limiting the availability of canine hosts at the population level [27, 30]. It should be noted, however, that data on the total number of dogs per municipality, including owned or registered dogs, were not directly available. Owned dogs are also susceptible to *Leishmania* infection and may contribute to the transmission cycle.

Second, the relationship between dog population management and human disease risk may reflect broader socioeconomic and public health infrastructure improvements. Areas with effective stray animal control programs often have better surveillance systems, leading to improved case detection and reporting of canine and human leishmaniasis [38].

The positive association between migrant populations and human VL incidence likely reflects multiple interconnected risk factors. Migration can contribute to VL incident cases through importation from other VL-endemic areas, including East Africa and the Indian subcontinent [39]. Surveillance data from 15 European centers between 2014 and 2019 showed that while 88% of VL cases were acquired in Europe, the epidemiology varies substantially by country. In Portugal, however, imported cases represent a negligible fraction of the total VL burden, with the vast majority being autochthonous transmission [13], suggesting that the association with migrant populations in this study reflects factors beyond disease importation.

These patterns may also reflect residual confounding or the ecological nature of the analysis, whereby associations at the municipality level do not necessarily correspond to individual-level relationships.

A similar association has been described for other poverty-related diseases in Portugal. In the case of tuberculosis, studies have shown links with social vulnerability and have reported that migrant populations in European countries may present higher incidence rates than populations in their countries of origin, with tuberculosis rates in recent migrants appearing up to 11 times more than in corresponding origin countries [40].

These factors likely include socioeconomic vulnerability and precarious living conditions characterized by poor housing quality, overcrowding, and limited access to preventive measures—all established risk factors for leishmaniasis transmission [41–43]. Housing characteristics play a critical role in creating favorable conditions for sand fly breeding and resting. Structures with mud walls, cracks, dampness, and darkness and proximity to animal sheds provide ideal microhabitats for phlebotomine vectors and significantly increase leishmaniasis risk [41, 44]. Migrant communities may disproportionately occupy substandard housing with these characteristics, increasing their exposure to sand fly vectors.

In addition, migrants often face barriers to accessing healthcare services [45], which may hinder timely diagnosis and management of immunosuppressive conditions that predispose to VL, such as advanced human immunodeficiency virus (HIV) infection/acquired immunodeficiency syndrome (AIDS). In Portugal, a higher proportion of migrant people from sub-Saharan Africa living with HIV are diagnosed at a later stage than native people [46]. More broadly, available data indicate that the burden of HIV infection is higher in migrant populations [47], and in Portugal, migrants account for a substantial proportion of newly diagnosed HIV cases [46]. Given that people living with HIV are known to be at increased risk of VL [48], migrants may be overrepresented within this particularly vulnerable group. This interpretation is also consistent with our Portuguese case series, in which approximately 20% of VL cases between 2010 and 2020 occurred in migrants from countries not considered endemic for leishmaniasis, among whom HIV infection was frequent [13], suggesting that immunosuppression may contribute to the clinical expression of disease in the migrant population.

Educational attainment demonstrates divergent associations with VL incidence versus seroprevalence in blood donors. In incidence models, higher proportions of residents with basic education or lower are associated with increased reported VL incidence. This finding aligns with education serving as a proxy for socioeconomic disadvantage and heightened vulnerability to clinical disease [42]. In addition, lower education can be associated with outdoor or informal employment increasing vector exposure [49], delayed healthcare-seeking behavior, and reduced health literacy [50]. Conversely, in seroprevalence models based on blood donors, higher proportions of residents with basic education or lower are associated with lower *Leishmania* seroprevalence. Blood donors represent a selected, generally healthier subgroup with higher health literacy and more stable healthcare engagement [51]. In municipalities with lower overall educational attainment, individuals with lower education may be underrepresented among blood donors owing to socioeconomic barriers, reduced voluntary donation participation, or exclusion related to comorbidities and occupational constraints. Consequently, seroprevalence estimates may disproportionately reflect exposure patterns of relatively more educated subpopulations, producing an apparent inverse association at the municipality level.

A higher seroprevalence of *Leishmania* in blood donors in Portugal was associated with a higher percentage of people living in localities with fewer than 2000 inhabitants at the municipality level possibly because rural and small-population settings typically have greater proximity to animal reservoirs, increased likelihood of peridomestic or outdoor exposure, and higher densities of competent sand fly vectors [52]. Similar associations between rurality and higher *Leishmania* infection rates have been observed in Spain, where small municipalities showed higher polymerase chain reaction (PCR) prevalence among blood donors [53].

Lower average altitude could be associated with higher seroprevalence because sand fly vectors are more abundant and active at lower elevations. Studies in the Mediterranean region have shown that sand fly density and *Leishmania* transmission decrease with increasing altitude, as cooler temperatures and short vector-activity seasons limit vector populations and transmission cycles [54, 55].

Lower average precipitation could be associated with higher seroprevalence because sand fly vectors prefer drier, warmer environments with lower relative humidity, which favor their development and activity. In Portugal, entomological studies have identified higher sand fly densities and increased *Leishmania* infection rates in areas with lower precipitation and higher temperatures, supporting the link between these climatic factors and increased transmission risk [56].

These findings have important implications for leishmaniasis control in Portugal and similar Mediterranean settings. Integrated control programs should emphasize vector control through topical insecticides (such as deltamethrin-impregnated collars), vaccination where available, and environmental management to reduce sand fly breeding sites [57, 58]. Targeted interventions for vulnerable populations, particularly migrant communities, should address the social determinants of leishmaniasis risk. This includes improving the quality of housing and ensuring access to preventive measures and healthcare in high-risk populations. Strengthening passive surveillance systems through improved reporting compliance and integration of electronic health records is fundamental to reduce underreporting and improve data quality for epidemiological monitoring. The One Health approach, coordinating human, animal, and environmental health surveillance, is essential for comprehensive understanding of transmission dynamics and effective disease control. Climate change adaptation strategies must be incorporated into long-term leishmaniasis control planning. Enhanced surveillance in areas projected to become climatically suitable for vector expansion, particularly in northern regions of Portugal, will enable early detection of changing transmission patterns. Predictive modeling using climate and environmental variables can identify priority areas for intervention and resource allocation. Future research should also include individual-level longitudinal cohort studies to overcome the limitations of ecological analyses and establish causal relationships between risk factors—such as housing characteristics and migration-related vulnerabilities—and infection/disease outcomes.

This study has several important limitations. The VL case data were obtained through a mandatory passive surveillance system (SINAVE), which is subject to substantial underreporting and depends heavily on healthcare access, diagnostic capacity, and reporting compliance. These factors may vary geographically across municipalities, potentially introducing systematic bias in the spatial distribution of reported cases [59]. The seroprevalence data were derived from blood donors, a convenience sample that differs systematically from the general population. Blood donors are required to meet strict health eligibility criteria that exclude individuals with acute or chronic illnesses—precisely the populations most likely to have symptomatic *Leishmania* infection [60]. In addition, the age restriction (18–65 years) excludes children and older adults. The ELISA test used also has inherent limitations for identifying asymptomatic infections. Although the manufacturer reports 97% sensitivity and 99% specificity, these performance characteristics are typically derived from patients with symptomatic VL and may not apply to asymptomatic individuals with lower antibody titers [61]. Anti-*Leishmania* antibodies can persist for years after resolved infection, meaning seropositivity does not necessarily indicate current active infection [62]. These findings should therefore be interpreted strictly as associations rather than causal effects. In multivariable models, individual coefficients may be influenced by correlations among covariates, conditioning on mediators, or adjustment for variables that do not act as true confounders. As such, the direction and magnitude of some associations, including seemingly paradoxical findings, may reflect model specification and underlying data structure rather than direct epidemiological relationships [63, 64]. The inability to control for individual-level confounders such as HIV status, specific housing conditions, occupational exposures, and behavioral risk factors also represents a major limitation [65]. Temporal limitations include the mismatch between VL case data (2010–2020) and seroprevalence data (2022), which reflect different epidemiological contexts. In addition, social and economic variables were primarily derived from the 2021 census and may not accurately represent conditions during earlier years of VL case occurrence.

## Conclusions

This study demonstrates that the distribution of *Leishmania infantum* infection in mainland Portugal is shaped by a complex interplay of environmental, climatic, and social determinants. While VL incidence was more strongly associated with indicators of social vulnerability, asymptomatic infection among blood donors was primarily linked to rurality and environmental conditions favorable to vector persistence. The divergent patterns observed between clinical disease and serological exposure highlight the influence of surveillance mechanisms, population selection, and access to healthcare on observed epidemiology.

These findings underscore the need for integrated, One Health-based control strategies that combine vector and reservoir management with targeted social and public health interventions. Strengthening surveillance and incorporating predictive modeling into national control programs will be essential to address current transmission patterns and anticipate future shifts under changing climatic conditions.

## Supplementary Information

Additional file 1: Supplementary Figure 1. Spatial distribution of social and economic variables across municipalities in mainland Portugal: a) Population density (in inhabitants/km2); b) Percentage of resident population living in localities with <2000 inhabitants; c) Percentage of male sex resident population; d) Percentage of resident population aged 0-14 years old; e) Percentage of resident population with basic education or lower; f) Percentage of unemployed resident population; g) Purchasing power per capita; h) Percentage of migrant population.

Additional file 2: Supplementary Figure 2. Spatial distribution of environmental and climatic variables across municipalities in mainland Portugal: a) Average altitude (in meters); b) Average precipitation per year (in millimeters); c) Average minimum winter temperature (in ºC); d) Average maximum summer temperature (in ºC); e) Percentage of forested area; f) Percentage of agricultural area; g) Average number of stray animals collected annually, between 2017 and 2021.

Additional file 3: Supplementary Figure 3. Spatial distribution of prediction uncertainty, expressed as the coefficient of variation, for: a) municipality-level mean VL incidence (2010–2020); b) blood-donor seroprevalence (2022).

Additional file 4: Supplementary Table 1. Full results of VL incidence.

Additional file 5: Supplementary Table 2. Full results of Leishmania seroprevalence.

## Data Availability

Data supporting the main conclusions of this study are included in the manuscript.

## References

[1] Manson’s Tropical Diseases. Elsevier; 2024. 10.1016/C2018-0-01574-1

[2] ECDC. Surveillance, prevention and control of leishmaniases in the European Union and its neighbouring countries [Internet]. 2022. https://www.ecdc.europa.eu/sites/default/files/documents/leishmaniasis-surveillance-eu.pdf

[3] Silva LP, Montenegro S, Werkauser R, Sales KGDS, Soares FCS, Costa VMA, Bezerra AC, Pinto MBDA, Ferreira SM, Neitzke-Abreu HC, Dantas-Torres F, Lima Junior MSDC. Asymptomatic Leishmania infection in blood donors from a major blood bank in Northeastern Brazil: a cross-sectional study. Rev Inst Med Trop Sao Paulo. 2020 Nov 27;62:e92. PMID: 33263698; PMCID: PMC7694538.10.1590/S1678-9946202062092. 33263698 10.1590/S1678-9946202062092 PMC7694538

[4] Aliaga L, Ceballos J, Sampedro A, Cobo F, López-Nevot MÁ, Merino-Espinosa G, et al. Asymptomatic *Leishmania* infection in blood donors from the Southern of Spain. Infection. 2019;47:739–47. 10.1007/S15010-019-01297-3. 30888587 10.1007/s15010-019-01297-3

[5] França ADO, Soares LS, Pompilio MA, Tozetti IA, Bonin CM, Dorval MEMC. Cytokine profile in *Leishmania*-positive blood donors. PLoS ONE. 2020;15:e0238933. 10.1371/journal.pone.0238933. 32966326 10.1371/journal.pone.0238933 PMC7511012

[6] Prevention EC for D, Control, Authority EFS. Phlebotomine sandflies maps. Stockholm: ECDC. 2023. https://ecdc.europa.eu/en/disease-vectors/surveillance-and-disease-data/phlebotomine-maps, Accessed 3 Mar 2024

[7] Maia C, Dantas-Torres F, Campino L. Parasite biology: the reservoir hosts. In: The Leishmaniases: Old Neglected Tropical Diseases. Springer International Publishing; 2018. p. 79–106. 10.1007/978-3-319-72386-0_4.

[8] Asfaram S, Fakhar M, Teshnizi SH. Is the cat an important reservoir host for visceral leishmaniasis? A systematic review with meta-analysis. J Venom Anim Toxins Incl Trop Dis. 2019 Jun 10;25:e20190012. PMID: 31258555; PMCID: PMC6583674. 10.1590/1678-9199-JVATITD-2019-0012. 31258555 10.1590/1678-9199-JVATITD-2019-0012 PMC6583674

[9] Molina R, Jiménez MI, Cruz I, Iriso A, Martín-Martín I, Sevillano O, et al. The hare (*Lepus granatensis*) as potential sylvatic reservoir of *Leishmania infantum* in Spain. Vet Parasitol. 2012;190:268–71. 10.1016/j.vetpar.2012.05.006. 22677135 10.1016/j.vetpar.2012.05.006

[10] Maia C, Conceição C, Pereira A, Rocha R, Ortuño M, Muñoz C, et al. The estimated distribution of autochthonous leishmaniasis by *Leishmania infantum* in Europe in 2005–2020. PLoS Negl Trop Dis. 2023;17:e0011497. 10.1371/journal.pntd.0011497. 37467280 10.1371/journal.pntd.0011497 PMC10389729

[11] Doenças de declaração obrigatória. Portal da Transparência, Serviços Partilhados do Ministério da Saúde, Lisboa, Portugal. 2018. https://transparencia.sns.gov.pt/explore/dataset/doencas-de-declaracao-obrigatoria/ Accessed 25 January 2026.

[12] Serrada E. A Leishmaniose visceral em Portugal Continental: 1999–2009 [PhD Thesis] [Internet]. Universidade Nova de Lisboa; 2010. http://hdl.handle.net/10362/5546

[13] Rocha R, Conceição C, Gonçalves L, Carvalho AC, Maia A, Martins A, et al. Epidemiological and clinical trends of visceral leishmaniasis in Portugal: retrospective analysis of cases diagnosed in public hospitals between 2010 and 2020. Infect Dis Poverty. 2024;13:41. 10.1186/s40249-024-01204-5. 38822396 10.1186/s40249-024-01204-5 PMC11143621

[14] Rocha R, Gonçalves L, Conceição C, Andrade P, Cristóvão JM, Condeço J, et al. Prevalence of asymptomatic *Leishmania* infection and knowledge, perceptions, and practices in blood donors in mainland Portugal. Parasit Vectors. 2023;16:357. 10.1186/s13071-023-05980-1. 37817278 10.1186/s13071-023-05980-1 PMC10563231

[15] Koch LK, Kochmann J, Klimpel S, Cunze S. Modeling the climatic suitability of leishmaniasis vector species in Europe. Sci Rep. 2017;7:13325. 10.1038/s41598-017-13822-1. 29042642 10.1038/s41598-017-13822-1 PMC5645347

[16] Chalghaf B, Chemkhi J, Mayala B, Harrabi M, Benie GB, Michael E, et al. Ecological niche modeling predicting the potential distribution of *Leishmania* vectors in the Mediterranean basin: impact of climate change. Parasit Vectors. 2018;11:461. 10.1186/s13071-018-3019-x. 30092826 10.1186/s13071-018-3019-x PMC6085715

[17] Wang D, Hof AR, Matson KD, Van Langevelde F, CLIMOS data providers, Dvořák V, et al. Understanding and predicting the geographic distributions of phlebotomine sand flies in and around Europe. Clim Change. 2025;178:205. 10.1007/s10584-025-04009-z. 41209429 10.1007/s10584-025-04009-z PMC12589297

[18] Carvalho BM, Maia C, Courtenay O, Llabrés-Brustenga A, Lotto Batista M, Moirano G, et al. A climatic suitability indicator to support *Leishmania infantum* surveillance in Europe: a modelling study. Lancet Reg Health - Eur. 2024;43:100971. 10.1016/j.lanepe.2024.100971. 39040529 10.1016/j.lanepe.2024.100971 PMC11261136

[19] Rodríguez-Escolar I, Balmori-de La Puente A, Collado-Cuadrado M, Bravo-Barriga D, Delacour-Estrella S, Hernández-Lambraño RE, et al. Analysis of the current risk of *Leishmania infantum* transmission for domestic dogs in Spain and Portugal and its future projection in climate change scenarios. Front Vet Sci. 2024;11:1399772. 10.3389/fvets.2024.1399772. 38756515 10.3389/fvets.2024.1399772 PMC11096601

[20] Franco AO, Davies CR, Mylne A, Dedet JP, Gállego M, Ballart C, et al. Predicting the distribution of canine leishmaniasis in Western Europe based on environmental variables. Parasitology. 2011;138:1878–91. 10.1017/S003118201100148X. 21914251 10.1017/S003118201100148X

[21] Santos FFM dos. O que são NUTS? PORDATA. https://www.pordata.pt/o+que+sao+nuts.

[22] Estatística IN de. Censos 2021 Resultados definitivos - Portugal [Internet]. 2022. https://www.ine.pt/ngt_server/attachfileu.jsp?look_parentBoui=586659861&att_display=n&att_download=y

[23] Saúde PDG da. SINAVE (Sistema Nacional de Vigilância Epidemiológica) [Internet]. https://www.dgs.pt/servicos-on-line1/sinave-sistema-nacional-de-vigilancia-epidemiologica.aspx

[24] Transplantação IIP do S e da. Relatório de Atividade Transfusional e Sistema Português de Hemovigilância 2022 [Internet]. 2023. https://www.hemovigilancia.net/files/RA_2022.pdf

[25] Pordata, Fundação Francisco Manuel dos Santos. Poder de compra per capita [Internet]. https://www.pordata.pt/sites/default/files/2024-06/Municipios_Poder-de-compra-per-capita.xlsx

[26] Direção Geral do Território. Modelos digitais do relevo [Internet]. 2025. https://www.dgterritorio.gov.pt/cartografia/cartografia-topografica/modelos-digitais

[27] Brun P, Zimmermann NE, Hari C, Pellissier L, Karger DN. CHELSA-BIOCLIM+ A novel set of global climate-related predictors at kilometre-resolution. EnviDat [data set]. 2022.. 10.16904/envidat.332

[28] Azevedo A, Peste F, Linck P, Carvalho J, Crawshaw D, Ferreira E, et al. Censo Nacional de Animais Errantes—2023, Relatório final. Departamento de Biologia & CESAM, Universidade de Aveiro. 2023 https://www.dgav.pt/wp-content/uploads/2025/07/Censo-Nacional-Animais-Errantes-2023.pdf

[29] Azevedo A, Peste F, Linck P, Carvalho J, Crawshaw D, Ferreira E, et al. Censo Nacional de Animais Errantes—2023. Relatório final. Aveiro: Departamento de Biologia & CESAM, Universidade de Aveiro.; 2023. p. 145pp.

[30] Bolker BM, Brooks ME, Clark CJ, Geange SW, Poulsen JR, Stevens MHH, et al. Generalized linear mixed models: a practical guide for ecology and evolution. Trends Ecol Evol. 2009;24:127–35. 10.1016/j.tree.2008.10.008. 19185386 10.1016/j.tree.2008.10.008

[31] Ospina R, Ferrari SLP. A general class of zero-or-one inflated beta regression models. Comput Stat Data Anal. 2012;56:1609–23. 10.1016/j.csda.2011.10.005.

[32] Brooks ME, Kristensen K, Benthem KJ, Magnusson A, Berg CW, Nielsen A, et al. glmmTMB balances speed and flexibility among packages for zero-inflated generalized linear mixed modeling. R J. 2017;9:378. 10.32614/RJ-2017-066.

[33] Naimi B, Hamm NAS, Groen TA, Skidmore AK, Toxopeus AG. Where is positional uncertainty a problem for species distribution modelling? Ecography. 2014 Feb;37(2):191–203. 10.1111/j.1600-0587.2013.00205.x. 30126638 10.1016/S0140-6736(18)31204-2

[34] Bongiorno G, Habluetzel A, Khoury C, Maroli M. Host preferences of phlebotomine sand flies at a hypoendemic focus of canine leishmaniasis in central Italy. Acta Trop. 2003;88:109–16. 10.1016/S0001-706X(03)00190-6. 14516922 10.1016/s0001-706x(03)00190-6

[35] Burza S, Croft SL, Boelaert M. Leishmaniasis. Lancet. 2018;392:951–70. 10.1016/S0140-6736(18)31204-2. 10.1016/S0140-6736(18)31204-2 30126638

[36] Staniek ME, Hamilton JGC. Odour of domestic dogs infected with Leishmania infantum is attractive to female but not male sand flies: evidence for parasite manipulation. Rowton E, editor. PLOS Pathog. 2021;17:e1009354. 10.1371/journal.ppat.1009354. 33735302 10.1371/journal.ppat.1009354 PMC7971543

[37] Chelbi I, Maghraoui K, Zhioua S, Cherni S, Labidi I, Satoskar A, et al. Enhanced attraction of sand fly vectors of Leishmania infantum to dogs infected with zoonotic visceral leishmaniasis. Barbosa DS, editor. PLoS Negl Trop Dis. 2021;15:e0009647. 10.1371/journal.pntd.0009647. 34314425 10.1371/journal.pntd.0009647 PMC8345872

[38] Bermudi PMM, Costa DNCC, Nunes CM, Tolezano JE, Hiramoto RM, Rodas LAC, et al. Canine serological survey and dog culling and its relationship with human visceral leishmaniasis in an endemic urban area. BMC Infect Dis. 2020;20:401. 10.1186/s12879-020-05125-0. 32503461 10.1186/s12879-020-05125-0 PMC7275440

[39] Di Muccio T, Scalone A, Bruno A, Marangi M, Grande R, Armignacco O, Gradoni L, Gramiccia M. Epidemiology of Imported Leishmaniasis in Italy: Implications for a European Endemic Country. PLoS One. 2015 Jun 26;10(6):e0129418. Erratum in: PLoS One. 2015 Jul 31;10(7):e0134885. PMID: 26114938; PMCID: PMC4482607. 10.1371/journal.pone.0134885. 26114938 10.1371/journal.pone.0129418 PMC4482607

[40] Domaszewska T, Koch A, Jackson S, Häcker B, Jonsson J, Langholz Kristensen K, Soini H, Arrazola de Oñate W, Guthmann JP, Hauer B, O Meara M, Nordstrand K, Sizaire V, de Vries G. Tuberculosis rates in migrants in low-incidence European countries, according to country of origin, reporting country and recency of immigration, 2014 to 2020. Euro Surveill. 2025 Mar;30(11):2400489. PMID: 40116030; PMCID: PMC1192707010.2807/1560-7917.ES.2025.30.11.2400489. 40116030 10.2807/1560-7917.ES.2025.30.11.2400489 PMC11927070

[41] Calderon-Anyosa R, Galvez-Petzoldt C, Garcia PJ, Carcamo CP. Housing characteristics and leishmaniasis: a systematic review. Am J Trop Med Hyg. 2018;99:1547–54. 10.4269/ajtmh.18-0037. 30382013 10.4269/ajtmh.18-0037 PMC6283488

[42] Valero NNH, Uriarte M. Environmental and socioeconomic risk factors associated with visceral and cutaneous leishmaniasis: a systematic review. Parasitol Res. 2020;119:365–84. 10.1007/s00436-019-06575-5. 31897789 10.1007/s00436-019-06575-5

[43] Heidari A, Dashtaki NM, Mizbani S, Rejali M, Maracy MR. Residential environment, human behavior and socio-economic status in transmission of cutaneous leishmaniasis in central Iran. Sci Rep. 2025;15:7271. 10.1038/s41598-025-91999-6. 40025204 10.1038/s41598-025-91999-6 PMC11873271

[44] Younis LG, Kroeger A, Joshi AB, Das ML, Omer M, Singh VK, et al. Housing structure including the surrounding environment as a risk factor for visceral leishmaniasis transmission in Nepal. Werneck GL, editor. PLoS Negl Trop Dis. 2020;14:e0008132. 10.1371/journal.pntd.0008132. 32150578 10.1371/journal.pntd.0008132 PMC7062236

[45] Dias S, Gama A, Cortes M, De Sousa B. Healthcare-seeking patterns among immigrants in Portugal: immigrants’ healthcare-seeking patterns. Health Soc Care Community. 2011;19:514–21. 10.1111/j.1365-2524.2011.00996.x. 21585582 10.1111/j.1365-2524.2011.00996.x

[46] Infeção por VIH em Portugal—2025. Lisboa: Portugal. Ministério da Saúde. Direção-Geral da Saúde/Instituto Nacional de Saúde Doutor Ricardo Jorge.; 2025.

[47] Santoso D, Asfia SKBM, Mello MB, Baggaley RC, Johnson CC, Chow EPF, et al. HIV prevalence ratio of international migrants compared to their native-born counterparts: a systematic review and meta-analysis. eClinicalMedicine. 2022;53:101661. 10.1016/j.eclinm.2022.101661. 36147629 10.1016/j.eclinm.2022.101661 PMC9486043

[48] Kantzanou M, Karalexi MA, Theodoridou K, Kostares E, Kostare G, Loka T, et al. Prevalence of visceral leishmaniasis among people with HIV: a systematic review and meta-analysis. Eur J Clin Microbiol Infect Dis. 2023;42:1–12. 10.1007/s10096-022-04530-4. 36427170 10.1007/s10096-022-04530-4 PMC9816214

[49] Abdullahi B, Mutiso J, Gicheru M. Social demographic characteristics associated with visceral leishmaniasis in West Pokot, Kenya. Am J Trop Med Hyg. 2024;110:930–5. 10.4269/ajtmh.23-0241. 38531111 10.4269/ajtmh.23-0241 PMC11066342

[50] Stormacq C, Van Den Broucke S, Wosinski J. Does health literacy mediate the relationship between socioeconomic status and health disparities? Integrative review. Health Promot Int. 2019;34:e1-17. 10.1093/heapro/day062. 30107564 10.1093/heapro/day062

[51] Bloch EM, Busch MP, Corash LM, Dodd R, Hailu B, Kleinman S, et al. Leveraging donor populations to study the epidemiology and pathogenesis of transfusion-transmitted and emerging infectious diseases. Transfus Med Rev. 2023;37:150769. 10.1016/j.tmrv.2023.150769. 37919210 10.1016/j.tmrv.2023.150769 PMC10841704

[52] Aliaga L, Ceballos J, Sampedro A, Cobo F, López-Nevot MÁ, Merino-Espinosa G, Morillas-Márquez F, Martín-Sánchez J. Asymptomatic Leishmania infection in blood donors from the Southern of Spain. Infection. 2019 Oct;47(5):739-747. Epub 2019 Mar 19. PMID: 30888587 10.1007/s15010-019-01297-3. 30888587 10.1007/s15010-019-01297-3

[53] Pérez-Cutillas P, Goyena E, Chitimia L, De La Rúa P, Bernal LJ, Fisa R, et al. Spatial distribution of human asymptomatic *Leishmania infantum* infection in Southeast Spain: a study of environmental, demographic and social risk factors. Acta Trop. 2015;146:127–34. 10.1016/j.actatropica.2015.03.017. 25800329 10.1016/j.actatropica.2015.03.017

[54] Prudhomme J, De Meeûs T, Toty C, Cassan C, Rahola N, Vergnes B, et al. Altitude and hillside orientation shapes the population structure of the *Leishmania infantum* vector *Phlebotomus ariasi*. Sci Rep. 2020;10:14443. 10.1038/s41598-020-71319-w. 32879357 10.1038/s41598-020-71319-w PMC7468129

[55] Alten B, Maia C, Afonso MO, Campino L, Jiménez M, González E, et al. Seasonal dynamics of phlebotomine sand fly species proven vectors of Mediterranean Leishmaniasis caused by *Leishmania infantum*. PLoS Negl Trop Dis. 2016;10:e0004458. 10.1371/journal.pntd.0004458. 26900688 10.1371/journal.pntd.0004458 PMC4762948

[56] Branco S, Alves-Pires C, Maia C, Cortes S, Cristovão JMS, Gonçalves L, et al. Entomological and ecological studies in a new potential zoonotic leishmaniasis focus in Torres Novas municipality, Central Region, Portugal. Acta Trop. 2013;125:339–48. 10.1016/j.actatropica.2012.12.008. 23262215 10.1016/j.actatropica.2012.12.008

[57] Dantas-Torres F, Miró G, Baneth G, Bourdeau P, Breitschwerdt E, Capelli G, et al. Canine leishmaniasis control in the context of One Health. Emerg Infect Dis. 2019;25:1–4. 10.3201/eid2512.190164. 31742505 10.3201/eid2512.190164 PMC6874277

[58] Gálvez R, Montoya A, Fontal F, Martínez De Murguía L, Miró G. Controlling phlebotomine sand flies to prevent canine *Leishmania infantum* infection: a case of knowing your enemy. Res Vet Sci. 2018;121:94–103. 10.1016/j.rvsc.2018.10.008. 30366124 10.1016/j.rvsc.2018.10.008

[59] Minter A, Medley GF, Hollingsworth TD. Using passive surveillance to maintain elimination as a public health problem for neglected tropical diseases: a model-based exploration. Clin Infect Dis. 2024;78:S169–74. 10.1093/cid/ciae097. 38662695 10.1093/cid/ciae097 PMC11088853

[60] O’Brien SF, Drews SJ, Lewin A, Russell A, Davison K, Goldman M, et al. How do we decide how representative our donors are for public health surveillance? Transfusion (Paris). 2022;62:2431–7. 10.1111/trf.17140. 10.1111/trf.17140 36193865

[61] Maritati M, Trentini A, Michel G, Hanau S, Guarino M, De Giorgio R, et al. Performance of five serological tests in the diagnosis of visceral and cryptic leishmaniasis: a comparative study. J Infect Dev Ctries. 2023;17:693–9. 10.3855/jidc.12622. 37279431 10.3855/jidc.12622

[62] Gidwani K, Picado A, Ostyn B, Singh SP, Kumar R, Khanal B, et al. Persistence of *Leishmania donovani* antibodies in past visceral leishmaniasis cases in India. Clin Vaccine Immunol. 2011;18:346–8. 10.1128/CVI.00473-10. 21159922 10.1128/CVI.00473-10 PMC3067357

[63] Diemer EW, Hudson JI, Javaras KN. More (adjustment) is not always better: how directed acyclic graphs can help researchers decide which covariates to include in models for the causal relationship between an exposure and an outcome in observational research. Psychother Psychosom. 2021;90:289–98. 10.1159/000517104. 34252900 10.1159/000517104 PMC8974490

[64] Westreich D, Greenland S. The Table 2 fallacy: presenting and interpreting confounder and modifier coefficients. Am J Epidemiol. 2013;177:292–8. 10.1093/aje/kws412. 23371353 10.1093/aje/kws412 PMC3626058

[65] Wakefield J. Ecologic studies revisited. Annu Rev Public Health. 2008;29:75–90. 10.1146/annurev.publhealth.29.020907.090821. 17914933 10.1146/annurev.publhealth.29.020907.090821

