# Supplementary figures and images for: Patterns and drivers of the distribution of Leishmania infection in humans using environmental and socioeconomic factors: a modeling study in mainland Portugal
Source: Parasit Vectors. 2026 May 22;19:291. doi: 10.1186/s13071-026-07451-9 (PMC13371380; doi:10.1186/s13071-026-07451-9)

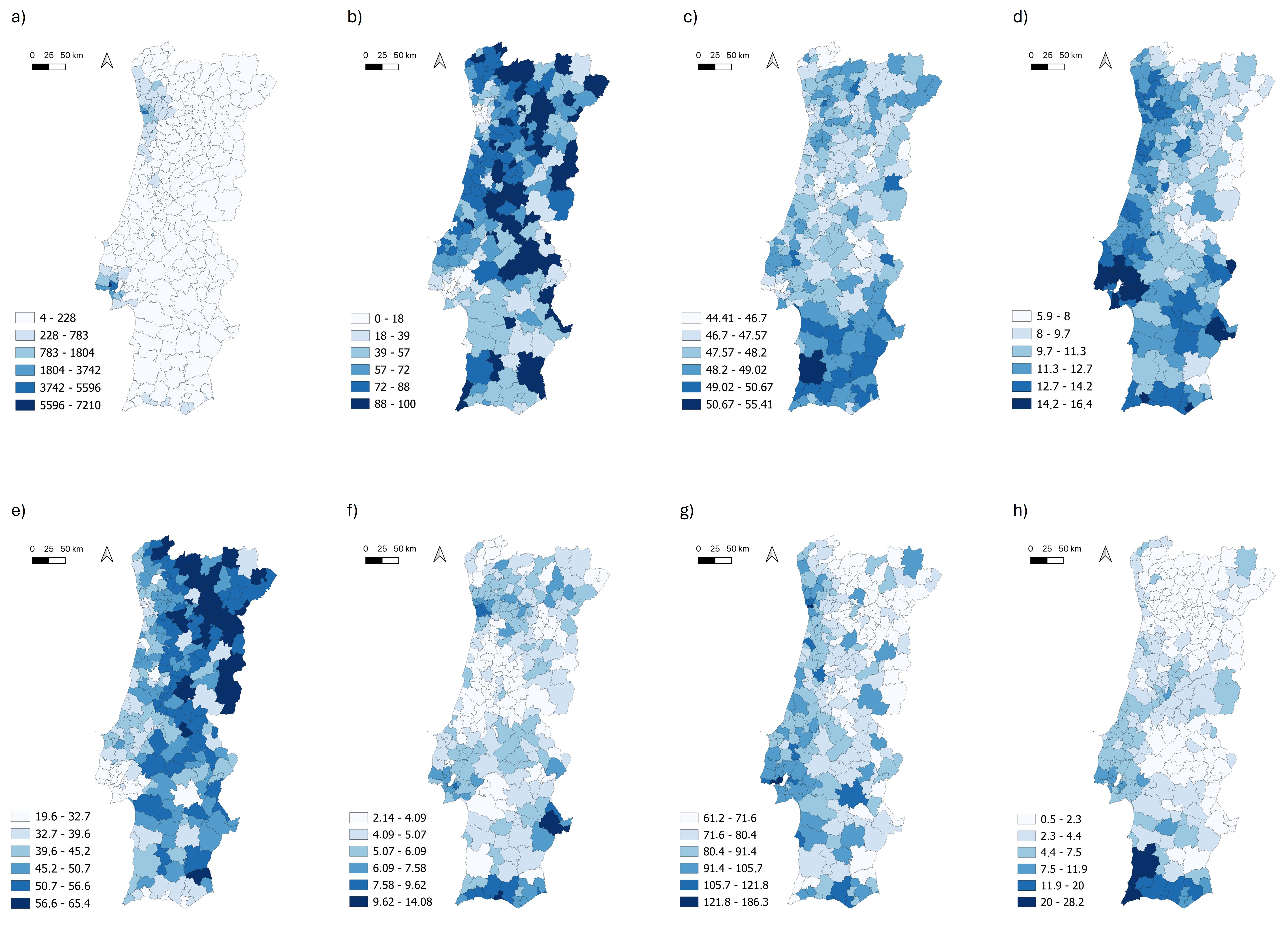

Supplement: Supplementary file 1 — Additional file 1: Supplementary Figure 1. Spatial distribution of social and economic variables across municipalities in mainland Portugal: a) Population density (in inhabitants/km2); b) Percentage of resident population living in localities with <2000 inhabitants; c) Percentage of male sex resident population; d) Percentage of resident population aged 0-14 years old; e) Percentage of resident population with basic education or lower; f) Percentage of unemployed resident population; g) Purchasing power per capita; h) Percentage of migrant population. [file 13071_2026_7451_MOESM1_ESM.jpg]

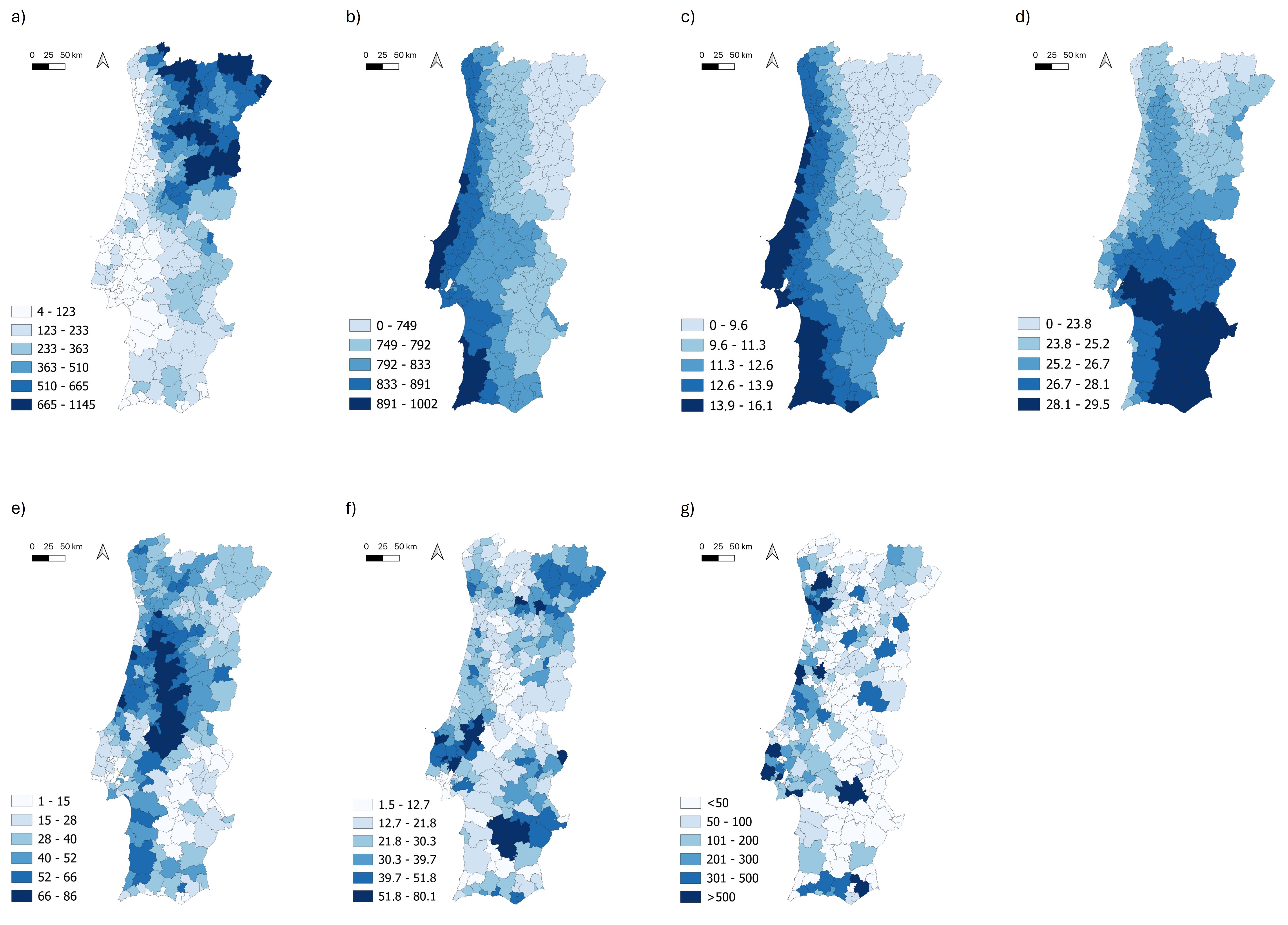

Supplement: Supplementary file 2 — Additional file 2: Supplementary Figure 2. Spatial distribution of environmental and climatic variables across municipalities in mainland Portugal: a) Average altitude (in meters); b) Average precipitation per year (in millimeters); c) Average minimum winter temperature (in ºC); d) Average maximum summer temperature (in ºC); e) Percentage of forested area; f) Percentage of agricultural area; g) Average number of stray animals collected annually, between 2017 and 2021. [file 13071_2026_7451_MOESM2_ESM.jpg]

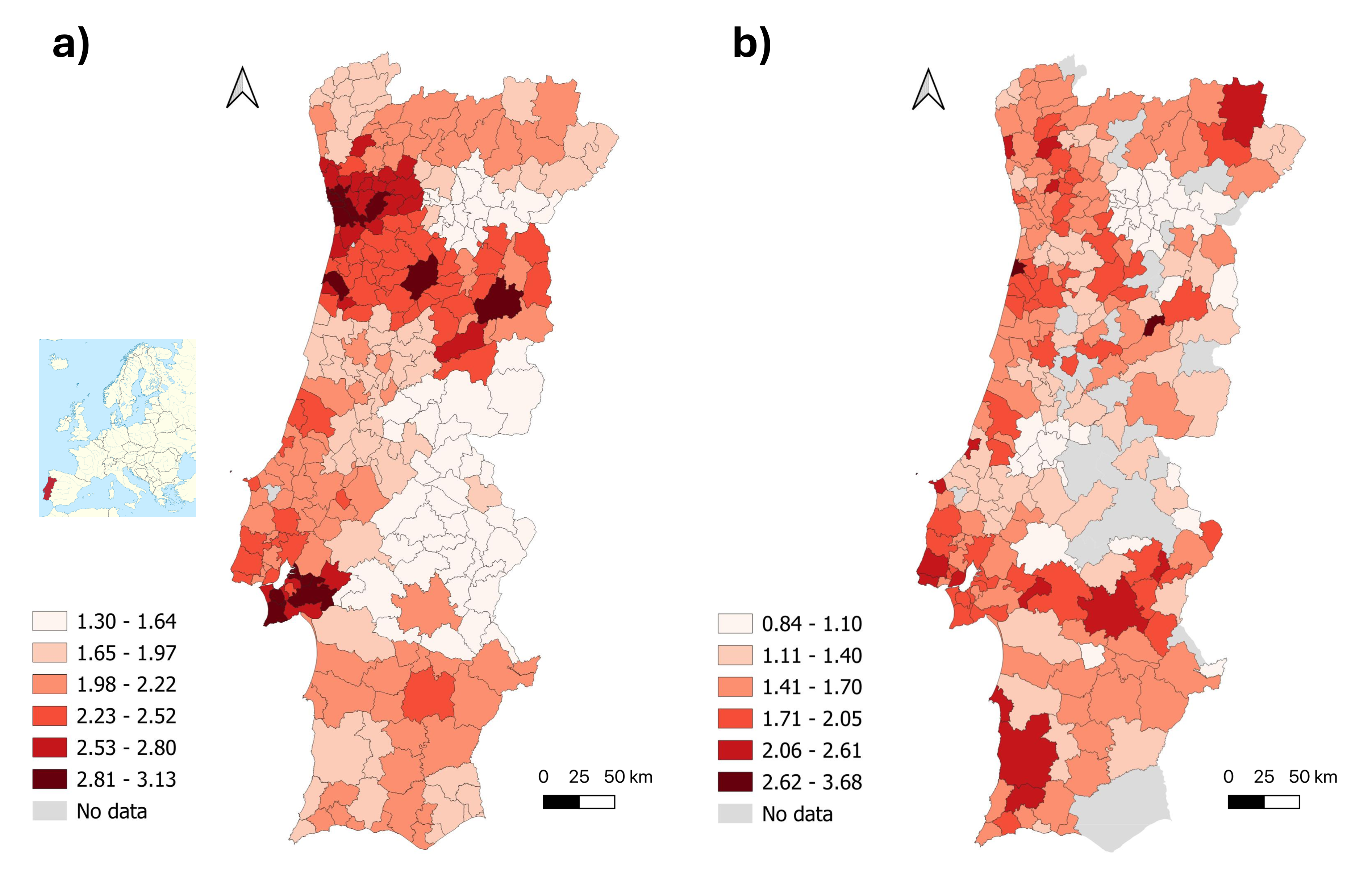

Supplement: Supplementary file 3 — Additional file 3: Supplementary Figure 3. Spatial distribution of prediction uncertainty, expressed as the coefficient of variation, for: a) municipality-level mean VL incidence (2010–2020); b) blood-donor seroprevalence (2022). [file 13071_2026_7451_MOESM3_ESM.png]
